# Oral Administration of the Probiotic *Lacticaseibacillus rhamnosus* CA15 in a Large Cohort of Women with Bacterial Vaginosis and Mixed Vaginitis: Clinical Evidence from a Randomized, Double-Blind, Placebo-Controlled Study

**DOI:** 10.3390/microorganisms13122651

**Published:** 2025-11-21

**Authors:** Alessandra Pino, Amanda Vaccalluzzo, Stefano Cianci, Marco Palumbo, Giuseppe Caruso, Cinzia Caggia, Cinzia L. Randazzo

**Affiliations:** 1 Department of Agriculture, Food and Environment, University of Catania, 95123 Catania, Italy; alessandra.pino@unict.it (A.P.); amanda.vaccalluzzo@unict.it (A.V.); ccaggia@unict.it (C.C.); 2 ProBioEtna SRL Spin off of the University of Catania, 95123 Catania, Italy; 3 Unit of Gynecology and Obstetrics, Policlinico “G. Martino”, Department of Human Pathology of Adult and Childhood “G. Barresi”, University of Messina, 98125 Messina, Italy; stefano.cianci@unime.it; 4 Department of General Surgery and Medical Surgical Specialties, Gynaecological Clinic, School of Medicine, University of Catania, 95123 Catania, Italy; mpalumbo@unict.it (M.P.); giu.caruso97@gmail.com (G.C.)

**Keywords:** probiotics, microbial dysbiosis, quality of life, women’s health, *Lacticaseibacillus rhamnosus*

## Abstract

Probiotics represent a valuable approach to boost vaginal health. A randomized double-blind placebo-controlled study was conducted to confirm the health benefits of the orally administered probiotic *Lacticaseibacillus rhamnosus* CA15 strain in a large cohort of women with bacterial vaginosis and mixed vaginitis, mainly related to mild aerobic vaginitis and vulvovaginal candidiasis. Recruited women were randomly assigned in a 1:1 ratio to receive, for 10 days, oral capsules containing 1 × 10^10^ colony-forming units of the *L. rhamnosus* CA15 strain (Active group) or a placebo (Placebo group). Two-hundred women completed this study. Clinical and microbiological parameters were assessed at baseline (T0), 10 days (T1), and 30 days post-treatment (T2). In addition, quality of life was evaluated at T0 and T2. The study protocol was registered on clinicaltrials.gov (ID: NCT05796921). The oral administration of the *L. rhamnosus* CA15 strain resulted in significant improvements in clinical characteristics of vaginal dysbiosis as well as changes in the vaginal microbiota composition. Furthermore, participants allocated to the Active group reported enhancements in physical and psychological health, social relations, environment, and overall quality of life. No significant changes were observed in the Placebo group. The present study highlights the ability of the *L. rhamnosus* CA15 to manage vaginal dysbiosis, offering new perspectives for the treatment and prevention of common gynecological disorders.

## 1. Introduction

Probiotics are widely recognized as health-promoting microorganisms that play a crucial role in preserving the health of the female reproductive tract, alleviating dysbiotic gynecological conditions, and boosting the vaginal immune response [1]. Advances in the study of vaginal microbiota composition and function have led to the widespread understanding that, under physiological conditions, this ecosystem is dominated by lactobacilli [2,3]. This group of bacteria is recognized as a key indicator of vaginal health, being able to exert protective effects, which are linked to the ability to produce antimicrobial compounds such as bacteriocins, bacteriocin-like inhibitory substances, organic acids, hydrogen peroxide (H_2_O_2_), siderophores, and biosurfactants [4]. Specifically, by hydrolyzing glycogen present in vaginal mucosal cells into glucose, and ultimately producing lactic acid, lactobacilli play a crucial role in restoring a healthy vaginal microbiome, since they are capable of inhibiting pathogen colonization and displacing pathogens already adhered to epithelial cells. Additionally, they act synergistically as a consortium in their probiotic activity [5,6,7]. A reduction in the proportion or abundance of lactobacilli is associated with various vaginal infections, including bacterial vaginosis (BV), vulvovaginal candidiasis (VVC), aerobic vaginitis (AV), and infections caused by human papillomavirus (HPV), human immunodeficiency virus (HIV), and herpes simplex virus 2 (HSV-2). Additionally, it is linked to other sexually transmitted infections (STIs) such as gonorrhea, trichomoniasis, *Chlamydia*, *Mycoplasma*, and *Ureaplasma* [8]. Among these, BV, VVC, and mixed vaginal infections are considered common problems in women [9,10]. Recent systematic reviews and meta-analyses have confirmed the potential benefits of probiotics in managing these dysbiotic conditions, including improvements in clinical outcomes, restoration of a balanced vaginal microbiota, symptom relief, and a reduction in infection recurrence rate. Additional benefits of probiotic lactobacilli include the reduction in genital inflammation and the preservation of the vaginal epithelial barrier integrity. Probiotic lactobacilli offer a valuable alternative or complementary approach to conventional antimicrobial therapies [11,12]. In this perspective, significant progress has been made in the development of supplements aimed at restoring the homeostasis of the vaginal microbiome [1,13,14]. Among probiotic lactobacilli, the *Lacticaseibacillus rhamnosus* CA15 strain (DSM 33960) has emerged as a valuable probiotic, capable of restoring and maintaining a balanced vaginal microbiota. Isolated from the vaginal ecosystem of a healthy woman, the *L. rhamnosus* CA15 strain (patent number IT202200016542A) was previously tested both in vitro and in vivo for its probiotic features [15,16]. Along with safety standards, the *L. rhamnosus* CA15 strain, demonstrated strong adhesion to both vaginal and intestinal cell lines, and the ability to antagonize a wide range of urogenital pathogens such as *Escherichia coli*, *Candida* spp., *Gardnerella vaginalis*, *Pseudomonas aeruginosa*, *Staphylococcus aureus*, and *Streptococcus agalactiae*. By measuring the scavenging activity of the superoxide radical generated by the self-oxidation of pyrogallol, it was found that the CA15 strain demonstrated antioxidant potential. This activity could be related to the production of bioactive metabolites able to mitigate oxidative stress in intestinal cells. In addition, the CA15 strain exerted anti-inflammatory activity, as revealed by a decrease in the IL-6 and, in turn, an increase in IL-2 in LPS-treated Caco-2 cells. This modulation indicates that the strain may influence immune responses by interacting with immune receptors, such as Toll-like receptors (TLRs), and activating signaling pathways that promote the production of anti-inflammatory cytokines. Due to these features, as well as its resilience under the harsh conditions of gastrointestinal passage, the *L. rhamnosus* CA15 was administered as a supplement in a randomized, double-blind, placebo-controlled trial involving 60 women with evident signs and symptoms of BV, complicated by an overgrowth of microorganisms related to mixed vaginitis (MV) [16]. After 10 days of the *L. rhamnosus* CA15 strain’s administration, significant improvements were observed in both clinical signs and symptoms of vaginal dysbiosis (burning, itching, leucorrhea, subjective vaginal discomfort, and vulvovaginal erythema/edema), a notable reduction in vaginal pathogens, and a concomitant increase in lactobacilli. These effects were observed even 30 days post-treatment. In addition, the *L. rhamnosus* CA15 strain’s administration led to significant improvements in the perceived physical health, social relations, and environment of the enrolled participants [16]. On this basis, the present randomized double-blind placebo-controlled trial aimed to confirm the effectiveness of the orally administered *L. rhamnosus* CA15 strain, in treating BV with co-occurring vaginal infections in a large cohort of women.

## 2. Materials and Methods

### 2.1. Trial Population

The present single-center randomized, double-blind, placebo-controlled trial was conducted at the Department of General Surgery and Medical Surgical Specialties, Gynaecological Clinic, AOU Policlinico G. Rodolico-San Marco Hospital, Catania (Italy). Participants with evident signs and symptoms of BV and co-occurring mixed vaginal infections voluntarily participated in this study without any financial compensation.

Inclusion criteria were being of fertile age (18–45 years); regular menstruation; presence of BV (at least 3 Amsel criteria; Nugent score ≥ 7); co-occurring AV (diagnosed based on the Donders’ score) and VVC (clinical picture and yeast culture); presence of at least one vaginal symptom (leucorrhea, burning, itching, erythema/edema or subjective vaginal discomfort); no participation in other clinical studies; consent to participate; willingness to collaborate in completing the study procedures; non-lactating status; appropriate personal hygiene; and the cognitive ability for collaboration. Participants with sexually transmitted diseases due to *Chlamydia*, *Neisseria gonorrheae*, or *Trichomonas vaginalis*; specific vaginitis related to acute AV and VVC; clinically apparent herpes simplex infection; precancerous lesions due to Human papillomavirus; human immunodeficiency virus infection; confirmed diagnosis of pelvic inflammatory disease (PID); recent use of antibiotic and/or antifungal drugs (less than one month); recent consumption of probiotics or food containing probiotics; recent use of immunosuppressive drugs (less than one month); pregnancy or breastfeeding; use of douching; hypersensitivity or allergy to any ingredient of investigational product or placebo; chronic diseases; neoplastic disease; diabetes; or genital tract bleeding were excluded from this study.

Information about the study protocol, procedures, and investigational product were provided to each participant, and they were assured of their right to withdraw from this study at any time. Written informed consent was obtained from all participants. All personal data were treated anonymously in accordance with Italian law, guaranteeing privacy.

### 2.2. Trial Design and Treatment

This study was conducted, from April 2023 to May 2025, according to the Good Clinical Practice and the World Medical Association (WMA) policy regarding Ethical Principles for Medical Research Involving Human Subjects, as stated in the Declaration of Helsinki. The study protocol was approved by the local Ethical Committee (registration number 163/2022/PO) and was registered on clinicaltrials.gov (ID: NCT05796921). All the design, analysis, interpretation of data, drafting, and revisions followed the CONSORT (CONsolidated Standards Of Reporting Trials) [17] and SPIRIT (Standard Protocol Items: Recommendation for Interventional Trials) Statements [18].

**Figure 1:**
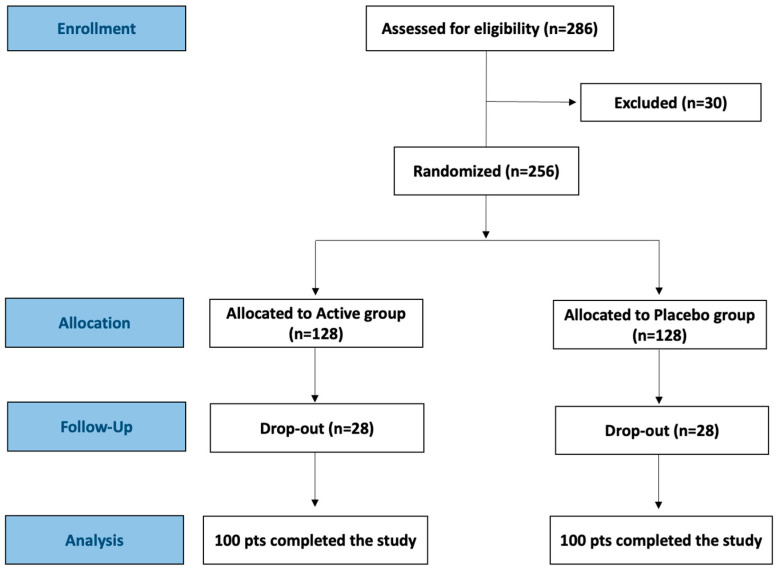
CONSORT flow diagram of this study.

The primary efficacy outcomes included changes in the composition of vaginal microbiota, with a significant reduction in potential pathogenic bacteria and an increase in lactobacilli, as well as attenuation or resolution of signs and symptoms of vaginal dysbiosis. The secondary outcome was the improvement of quality of life (QoL). A priori power analysis was conducted to determine the appropriate sample size for detecting a clinically meaningful difference in the primary efficacy outcomes between Active and Placebo groups at the 30-day post-treatment evaluation (T2). Based on preliminary data from a pilot trial involving 60 participants [16], an effect size (Cohen’s d) of 0.6 was estimated for between-group differences in microbiological and clinical outcome scores. To achieve a power of 0.90 at an alpha level of 0.05 (two-tailed), a minimum sample size of 86 participants per group was required. Accounting for an anticipated dropout rate of 15–20%, to ensure sufficient power and maintain statistical validity across all primary and secondary endpoints, a total of 286 participants were assessed for eligibility and 256 of them were enrolled. Following informed consent, enrolled participants were randomly assigned in a 1:1 ratio to receive either the Active or Placebo treatment, as illustrated in the CONSORT flow diagram (Figure 1). Randomization was implemented using a computer-generated sequence via a centralized, permuted block method with variable block sizes, ensuring allocation concealment. The randomization list was generated by an independent statistician not involved in the clinical aspects of this study. Allocation assignments were concealed in opaque, sequentially numbered envelopes and maintained by study personnel uninvolved in data collection or outcome assessment. Participants took one capsule per day for 10 consecutive days. The Active treatment consisted of a transparent vegetable capsule containing the *L. rhamnosus* CA15 strain and corn starch. The CA15 strain, belonging to the culture collection of ProBioEtna srl (spinoff of the University of Catania), was isolated from the vaginal ecosystem of a healthy Italian woman, of reproductive age, at the Department of General Surgery and Medical Surgical Specialties, General Hospital G. Rodolico, University of Catania (Catania, Italy), who was recruited to an observational study approved by the Local Ethical Committee (n. 16/2022/PO). The Active treatment contained at manufacturing 15 billion viable cells while, after 18 months of storage at room temperature (25 ± 2 °C), the final cell density was 5 billion viable cells. The coefficients of variation were 0.11 and 0.43, respectively. The placebo capsules were identical in appearance and composition, except for the absence of the bacterial strain. Capsules, manufactured by SYNBIOTEC srl (Camerino, Italy), were provided in standardized blisters of 15 units. Blinding was maintained for participants, investigators, laboratory personnel, and data analysts throughout the study duration.

### 2.3. Visits and Assessment

After the baseline assessment (T0), follow-up visits were conducted 10 days (T1) and 30 days post-treatment (T2). At baseline, demographic data were collected, including information on age, body mass index (BMI), smoking habits, contraceptive use, history of vaginal infection, and sexual activity. At each sampling time (T0, T1, and T2), clinical signs and symptoms, Amsel criteria (homogenous vaginal discharge, presence of clue cells, positive amine test, and vaginal fluid pH) [19], Nugent score [20], lactobacillary grade (LBG), and vaginal microbiota composition were evaluated.

#### 2.3.1. Clinical Signs and Symptoms

The clinical signs and symptoms—leucorrhea (associated with both BV and VVC); burning, itching, and vulvovaginal erythema/edema (associated with VVC); discharge and subjective vaginal discomfort (mainly associated with AV)—were assessed on a severity scale ranging from 0 (absent or normal) to 3 (severe). The Nugent score was assessed using a 10-point scale based on microscope examination (1000× magnification, oil immersion) of Gram-stained vaginal smears. A score between 0 and 3, characterized by the dominance of lactobacilli, was considered as normal vaginal microbiota, a score of 4–6 was classified as intermediate, and a score of 7–10, dominated by Gram-negative and Gram-variable rods, was presumed to be a BV-like condition [20]. The lactobacillary grade (LBG) was evaluated according to the Donders classification, with LBG I indicating normal microbiota dominated by lactobacilli, LBG II indicating a reduced number of lactobacilli mixed with other bacteria, and LBG III indicating the absence of lactobacilli and the presence of other bacteria [21].

#### 2.3.2. Microbiological Analysis of Vaginal Samples

The composition of the vaginal microbiota was evaluated at each sampling time (T0, T1, and T2) by plate count. The limits of detection and of quantification (LoD and LoQ, respectively) were determined according to ISO 7218:2024 [22]. In detail, the LoD correspond to one colony per plated volume (1 mL), and the LoQ was defined as 30 cfu/mL. Vaginal swabs were collected from the lateral vaginal wall and the posterior vaginal fornix using sterile cotton-tipped swabs, filled with the transport medium Transystem Amies Clear (Biolife, Milan, Italy), and were immediately transferred, under refrigerated conditions, to the Laboratory of ProBioEtna, a spinoff of the University of Catania (Catania, Italy). Swabs were subjected to microbiological counting through 10-fold dilutions, which were plated using the following agar media and conditions: Rogosa SL agar (Biolife, Milan, Italy) for *Lactobacillus* counts, incubated at 35–37 °C for 40–48 h; Columbia Blood Agar base (Oxoid, Milan, Italy), supplemented with Gardnerella Vaginalis Selective Supplement (Oxoid, Milan, Italy), incubated at 37 °C for 40–48 h for *Gadnerella* spp. count; MacConkey Agar Mug (Biolife, Milan, Italy) incubated at 37 °C for 16–18 h for *Escherichia coli*; Mannitol Salt Agar (Oxoid, Milan, Italy) incubated at 32 °C for 48 h for the count of staphylococci; Slanetz Bartley Agar (Biolife, Milan, Italy) incubated at 37 °C for 48 h for enterococci; and Chromogenic Candida Agar (Biolife, Milan, Italy), incubated at 35–37 °C for 18–48 h, for the count of *Candida* spp.

The microbiological count was performed in triplicate. Results were expressed as mean log_10_ cfu/mL, and standard deviation was calculated to assess measurement repeatability.

#### 2.3.3. Quality of Life

The quality of life (QoL) was evaluated, both at baseline (T0) and 30 days post-treatment (T2), using the World Health Organization Quality of Life-BREF (WHOQOL-BREF) questionnaire [23]. The questionnaire includes 26 items, 24 of which assess four domains of the quality of life (physical health, psychological health, social relationships, environment) while the remaining two items, examined separately, are related to the individual’s perception of overall quality of life and general health. Responses to each item are given on a 1–5 Likert-type scale, where 1 represents the lowest level of agreement and 5 indicates the highest level of agreement with a given statement. The scores for each of the four domains were converted into a scale ranging from 0 to 100 and expressed as means. Higher mean scores reflect a better perception of QoL.

### 2.4. Statistical Analysis

The Mann–Whitney U test was applied to evaluate baseline differences between Active and Placebo groups for variables such as age, BMI, Nugent score, and LBG data. Baseline characteristics related to smoking habits, contraceptive use, history of vaginal dysbiosis, sexual activity, vulvovaginal signs and symptoms, and presence of Amsel criteria were analyzed using Fisher’s exact test. To assess intra-group changes over time in vulvovaginal signs and symptoms, Nugent score, LBG data, QoL, and vaginal microbiota composition, the Wilcoxon Signed-Rank Test was applied. Amsel criteria data were evaluated using the non-parametric McNemar test, specifically suited for paired nominal data, to detect differences within groups over time. Inter-group differences in vulvovaginal signs and symptoms, Nugent score, LBG data, quality of life, and vaginal microbiota composition were assessed through the Analysis of Covariance (ANCOVA), with baseline values as covariates. For inter-group comparisons related to Amsel criteria, logistic regression analysis was performed after baseline normalization. All statistical analyses were conducted using the SciPy library in Python (version 3.9.19, SciPy 1.13.1) and differences were considered statistically significant at *p*-value < 0.05. *p*-values were corrected using the Benjamini–Hochberg method.

## 3. Results

### 3.1. Participant Flow

A total of 286 women, who met the inclusion and exclusion criteria, were initially assessed for eligibility. Among these, 10 were excluded for unconfirmed microbiological diagnosis of BV and of mixed vaginal infections and 20 declined to participate. The remaining participants (n = 256) were randomly assigned in a 1:1 ratio, to either the Active (n = 128) or Placebo group (n = 128). During the follow-up visits, scheduled 10 days (T1) and 30 days (T2) post-treatment, 56 participants dropped out for different reasons (lost to follow-up, discontinued adherence to the therapeutic regime, fever for seasonal flu, pregnancy, and others). A per-protocol analysis was performed including only participants who adhered fully to the intervention protocol. No severe adverse events were observed in either group. Table S1 reports the adverse events, capsule adherence, concomitant medications, and pregnancy screening. In detail, mild abdominal pain, nausea, and flatulence were reported in both groups with no statistically significant differences (*p* > 0.05). No pregnancies occurred among participants during the trial.

### 3.2. Demographic and Baseline Clinical Characteristics

The baseline demographic and clinical characteristics of the enrolled subjects are reported in Table 1 and Table 2, respectively. Overall, the two groups were homogeneous in terms of demographic characteristics and baseline clinical data. The mean ages of participants assigned to the Active and Placebo groups were 32.58 ± 5.74 and 32.13 ± 6.52, respectively (*p*-value = 0.7727). The majority of the participants had a BMI within the healthy normal range (between 18.5 and 24.9), were sexually active (Active, n = 68; Placebo, n = 71), were contraceptive users (Active, n = 76; Placebo, n = 79), and had a history of vaginal dysbiosis (Active, n = 61; Placebo, n = 58). Only 24 subjects allocated to the Active group and 22 allocated to the Placebo group had smoking habits (Table 1). Concerning clinical characteristics (Table 2), leucorrhea, burning, and subjective vaginal discomfort were the most reported symptoms in both groups. Although all participants satisfied at least three Amsel criteria, the presence of homogeneous vaginal discharge and a vaginal pH > 4.5 were the most frequently observed in both groups. Almost all participants had a Nugent score between 7 and 10 (Active, n = 97; Placebo, n = 95) and presented LBG III (Active, n = 92; Placebo, n = 89).

Data related to age are presented as means ± SD. All the other characteristics are reported as numbers of patients. Statistical significance between Active and Placebo groups was set at *p* < 0.05.

Data are reported as the number of patients. Statistical significance between Active and Placebo groups was set at *p* < 0.05.

### 3.3. Effect of Probiotic and Placebo Treatment on Diagnostic Parameters

#### 3.3.1. Clinical Signs and Symptoms

**Figure 2:**
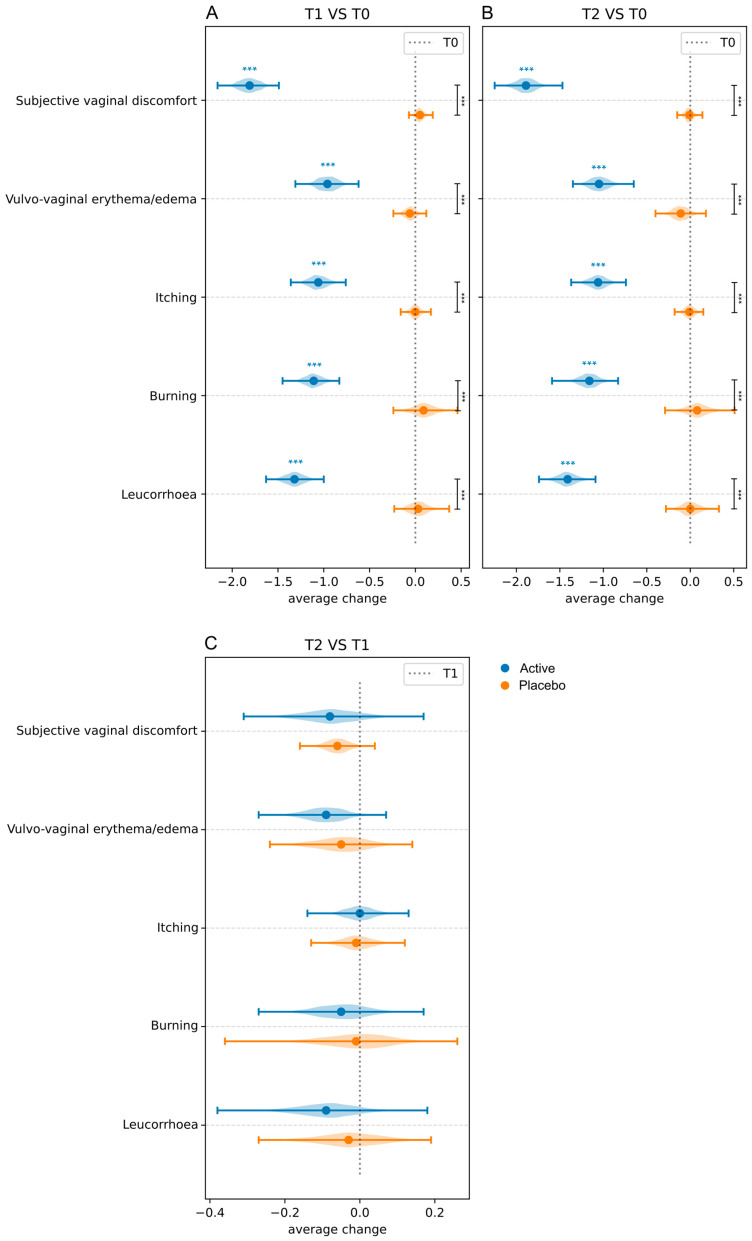
Average changes in clinical signs and symptoms in both Active and Placebo groups. Intra-group comparisons (T1 vs. T0) and inter-group comparisons at T1 (**A**); intra-group comparisons (T0 vs. T2) and inter-group comparisons at T2 (**B**); intra-group comparisons (T2 vs. T1) (**C**). All inter-group comparisons were performed after baseline (T0) normalization. Blue and black stars are related to intra- and inter-group comparisons, respectively. *** = *p* ≤ 0.001.

Data on clinical signs and symptoms (leucorrhea, burning, itching, vulvovaginal erythema/edema, and subjective vaginal discomfort), evaluated using a three-point severity score (0, absent or normal; 3, severe) in both Active and Placebo groups at baseline (T0) and 10 days (T1) and 30 days post-treatment (T2), as well as intra-group and inter-groups differences, are displayed in Figure 2 and detailed in Tables S2 and S3.

Concerning intra-group differences, compared to baseline, a statistically significant reduction in the mean severity score for all signs and symptoms was observed in the Active group both 10 days and 30 days post-treatment. In contrast, no statistically significant changes were observed in the Placebo group throughout this study.

Results on the differences between the Active and Placebo groups (inter-group differences) are reported in Figure 2 and detailed in Table S3. Statistically significant differences (*p* < 0.05) were observed for all the signs and symptoms evaluated, indicating that the Active group experienced significantly greater changes in symptoms from baseline compared to the Placebo group.

#### 3.3.2. Amsel Criteria

**Figure 3:**
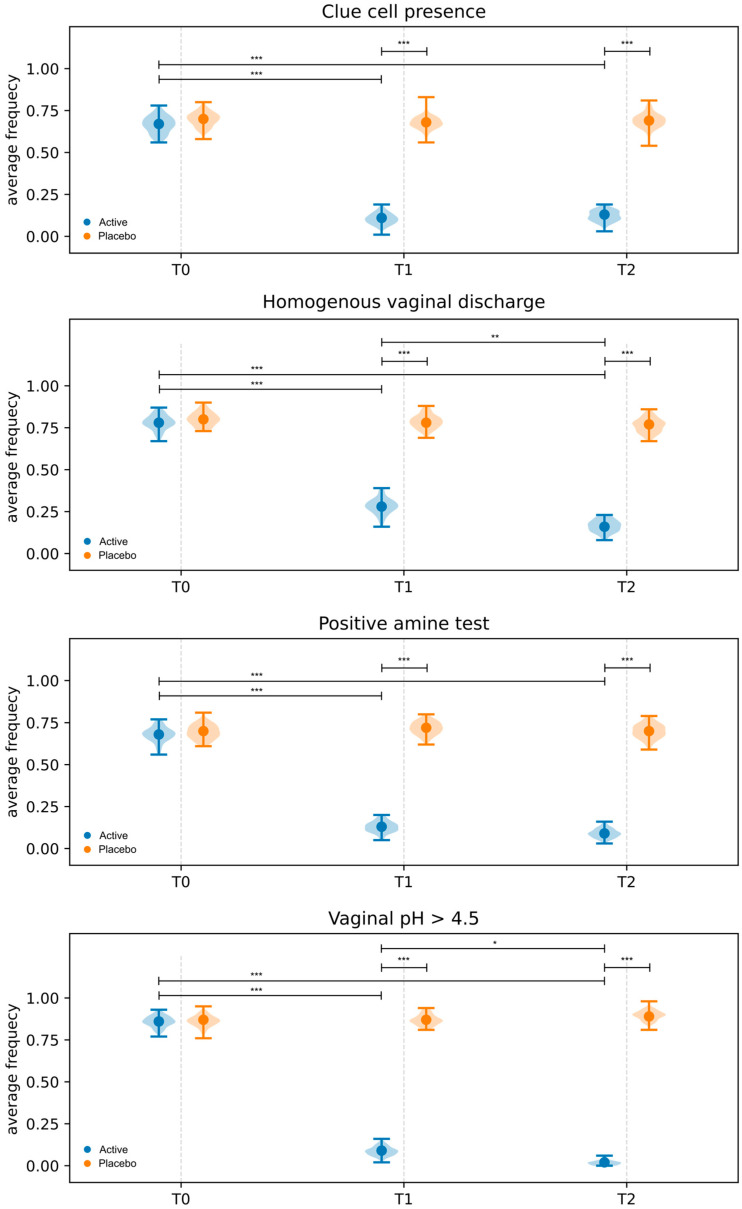
Amsel criteria evaluated in both Active and Placebo groups at baseline (T0), 10 days (T1), and 30 days (T2) after the end of the treatment. Data are reported as average frequency. Intra-group comparison and inter-group comparisons, after baseline (T0) normalization, are displayed. * = *p* ≤ 0.05, ** = *p* ≤ 0.01, *** = *p* ≤ 0.001.

All subjects in the Active and Placebo groups met at least three Amsel criteria at baseline. Intra-group and inter-group differences were evaluated using the McNemar test and logistic regression, respectively. As shown in Figure 3 and Table S4, statistically significant intra-group changes were observed only in the Active group. In fact, compared to baseline, a reduction in the average frequency of homogenous vaginal discharge, clue cells, positive amine test, and vaginal pH higher than 4.5 was revealed at both T1 and T2 sampling times. Interestingly, compared to T1, a statistically significant reduction in the average frequency of homogenous vaginal discharge (*p* = 0.0059) and vaginal pH > 4.5 (*p* = 0.0455) was revealed at T2 (Table S4). In contrast, no statistically significant changes were observed in the Placebo group throughout this study (Figure 3, Table S4). Concerning the inter-group analysis, after baseline normalization, statistically significant differences (*p* < 0.05) were detected between Active and Placebo groups at both T1 and T2 sampling times (Figure 3; Table S5). Based on the treatment effect, a significant reduction in the number of participants meeting the Amsel criteria was observed only in the Active group.

#### 3.3.3. Nugent Score and Lactobacillary Grade

**Figure 4:**
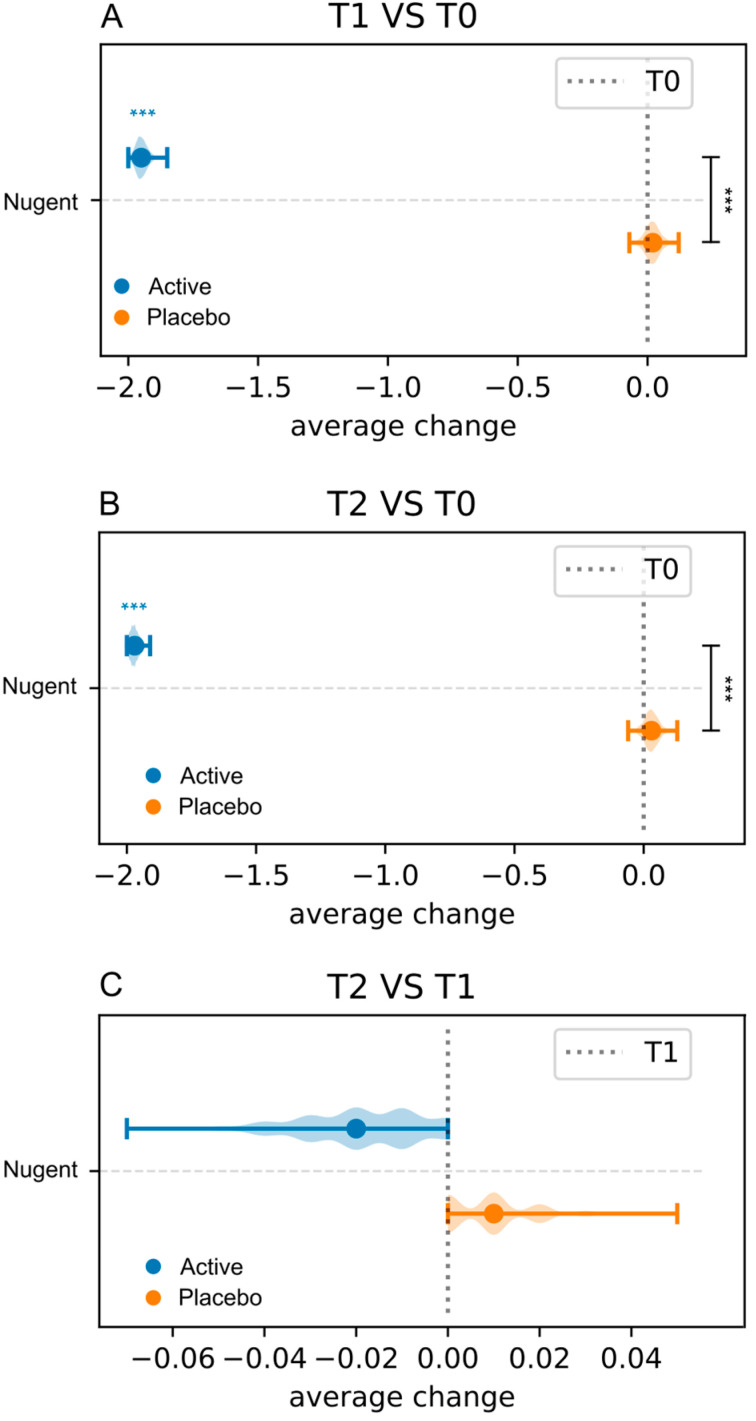
Intra-group and inter-group comparison of Nugent scores. Data are reported as average change. Intra-group comparison (T0 vs. T1) and inter-group comparisons at T1 (**A**); intra-group comparisons (T0 vs. T2) and inter-group comparisons at T2 (**B**); intra-group comparisons (T2 vs. T1) (**C**). Blue and black stars are related to intra- and inter-group comparisons, respectively. *** = *p* ≤ 0.001.

**Figure 5:**
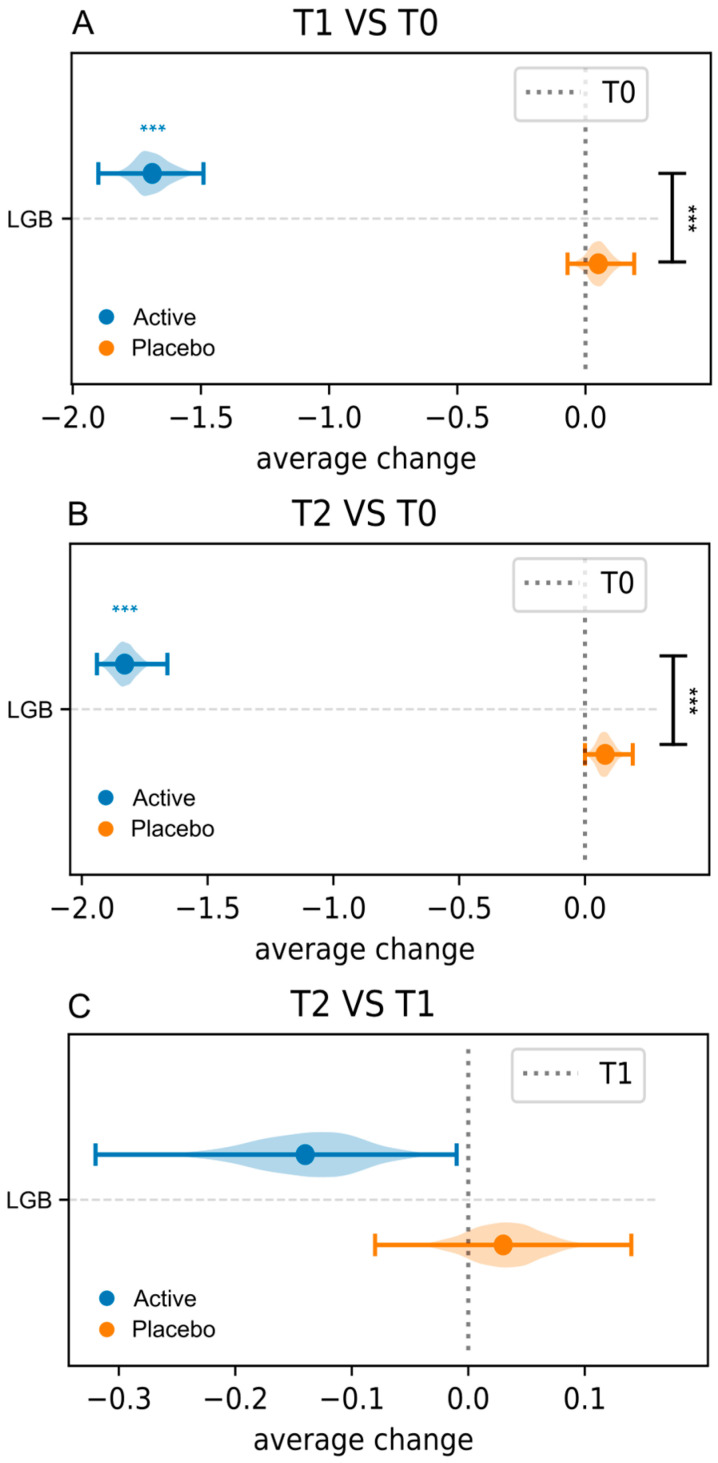
Intra-group and inter-group comparison of LBG data. Data are reported as average change. Intra-group comparisons (T0 vs. T1) and inter-group comparisons at T1 (**A**); intra-group comparisons (T0 vs. T2) and inter-group comparisons at T2 (**B**); intra-group comparisons (T2 vs. T1) (**C**). Blue and black stars are related to intra- and inter-group comparisons, respectively. *** = *p* ≤ 0.001.

Data on the Nugent score are displayed in Figure 4 and in Table S6. The Wilcoxon Signed-Rank Test was applied to assess intra-group variations across sampling times (T0, T1, and T2). In the Active group, compared to baseline, a significant reduction in the Nugent score was observed both at T1 and T2, and no subjects had a Nugent score between 7 and 10. Differently, no statistically significant changes were observed in the Placebo group throughout this study (Figure 4, Table S6). After baseline normalization, statistically significant inter-group differences were revealed at both T1 and T2 sampling times (Figure 4, Table S6). Data on the LBG are shown in Figure 5 and in Table S7. Statistically significant intra-group differences were observed only in the Active group at both T1 and T2 sampling times compared to baseline (Figure 5, Table S7).

#### 3.3.4. Effect of Probiotic and Placebo Treatment on the Vaginal Microbiota Composition

The composition of the vaginal microbiota of participants, allocated to Active and Placebo groups, was evaluated at T0, T1, and T2 sampling times by the plate count, and the results, expressed as mean log_10_ cfu/mL and standard deviation, are reported in Table 3. Overall, at baseline (T0), participants of both Active and Placebo groups showed a dysbiotic microbiota, characterized by an overabundance of pathogenic bacteria and a reduced presence of lactobacilli. In comparison to T0, a statistically significant reduction in all pathogens and a significant increase in lactobacilli were achieved in the Active group at both T1 and T2 sampling times (Table 3), revealing the ability of the *Lacticaseibacillus rhamnosus* CA15 strain to maintain a balanced vaginal microbiota till 30 days post-treatment. In contrast, the Placebo group showed a different trend, with a vaginal microbiota imbalance throughout this study. The observed statistically significant changes were related to an increase in the cell density of almost all the investigated potential pathogens and a weak increase in lactobacilli (Table 3). As reported in Table 4, the ANCOVA revealed inter-group differences, at both T1 and T2 sampling times, for all the investigated microbial groups, demonstrating that only the treatment was associated with significant beneficial changes in the vaginal microbiota composition.

### 3.4. Effect of Probiotic and Placebo Treatment on the Perceived Quality of Life

**Figure 6:**
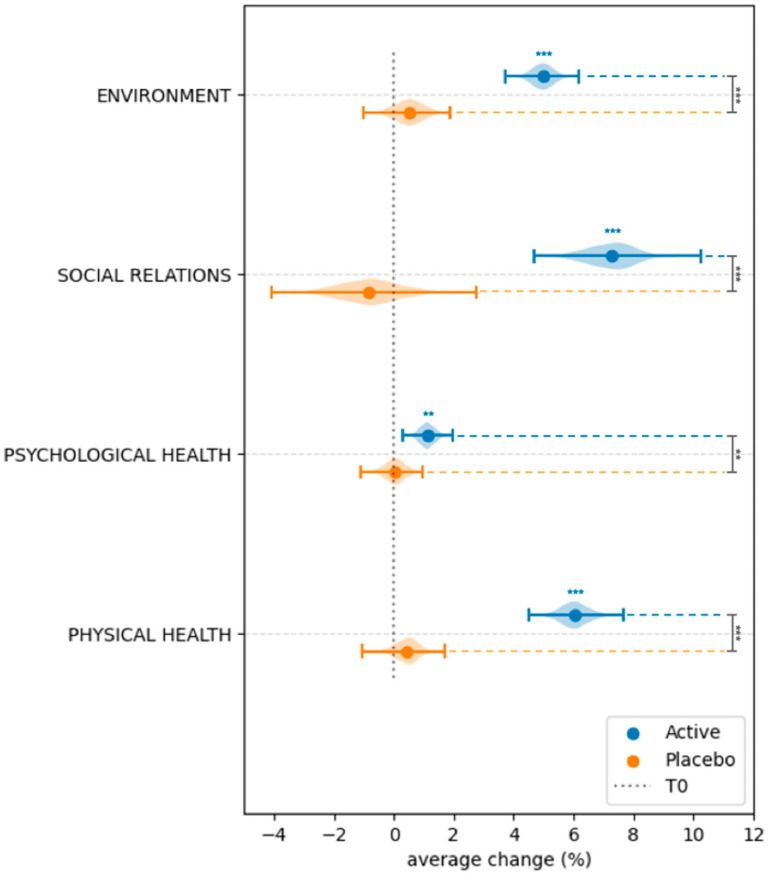
Intra-group and inter-group comparison of physical health, psychological health, social relations, and environment domains of the quality of life. Data are reported as average change. Blue and black stars are related to intra- and inter-group comparisons, respectively. ** = *p* ≤ 0.01, *** = *p* ≤ 0.001.

**Figure 7:**
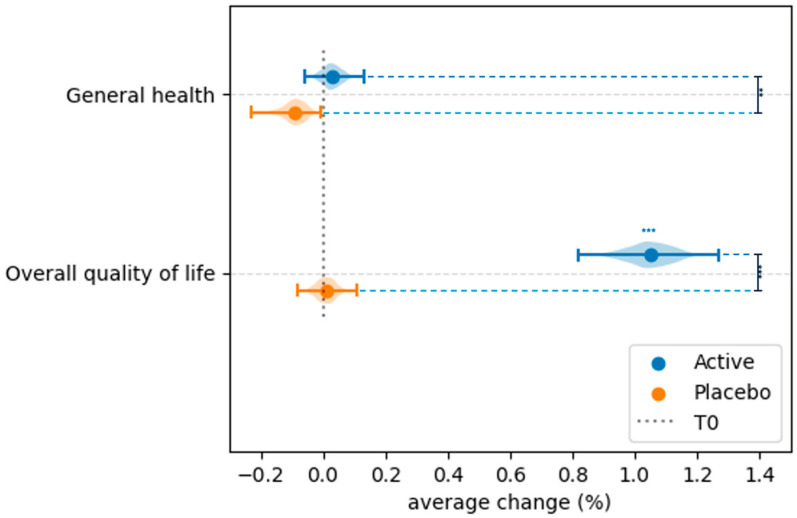
Intra-group and inter-group comparison of general health and overall quality of life domains of the quality of life. Data are reported as average change. Blue and black stars are related to intra- and inter-group comparisons, respectively. ** = *p* ≤ 0.01, *** = *p* ≤ 0.001.

Results on the perceived QoL, assessed at baseline and 30 days post-treatment, using the WHOQOL-BREF questionnaire, are reported in Figure 6 and Figure 7 and in Table S8. Compared to baseline, the Wilcoxon Signed-Rank Test revealed statistically significant changes in all the QoL domains in the Active group (physical health, psychological health, social relations, environment) (Figure 6). In addition, a significant improvement in the overall perception of quality of life (*p* = 4.73 × 10^−17^) was shown (Figure 7, Table S8). In comparison, among the different domains, no significant variations were detected in the Placebo group (Figure 6 and Figure 7, Table S8). The ANCOVA, used to compare the treatment effect between the Active and Placebo groups after baseline normalization, showed significant differences in all the QoL domains (Table S8).

## 4. Discussion

The imbalance of the vaginal microbiota, frequently accompanied by symptoms that cause discomfort, represents the main cause of vaginal dysbiosis, with BV, VVC, AV, and mixed vaginal infections being the most common conditions [24]. Antifungal and antibiotic treatments generally used in clinical practice, although selective against target pathogens, can suppress beneficial lactobacilli, causing a vaginal pH increase and recurrent rate of infection [25]. Hence, the use of probiotic lactobacilli has emerged as an alternative or supplementary therapy for vaginal infections, garnering increasing interest from both clinicians and patients dealing with vaginal disorders [26]. Both vaginal and oral administration routes have been explored. Research investigating the underlying mechanism by which oral probiotics impact the vaginal microbiota hypothesizes that orally administered probiotics can translocate from the rectum to the vagina, thereby promoting specific clinical effects (e.g., via alleviating dysbiosis). Moreover, emerging studies suggest that the gastrointestinal microbiota is interconnected with the vagina through the “vagina–gut axis,” which facilitates a bidirectional microbial translocation [25,27,28]. In this context, several hypotheses have been put forward about the ascending pathway, as the rectum can act as a reservoir for vaginal lactobacilli and facilitate microbial exchange between adjacent sites, significantly impacting the vaginal health [27,28,29]. In addition to bidirectional translocation, indirect interactions between the vagina and gut, through the hematogenous route, lymph node transfer, or hormonal pathways, have been suggested [27,29]. These pathways are not mutually exclusive, and future studies, combining longitudinal fecal, rectal, vaginal, and systemic sampling, will be necessary to clarify the dynamics of this process.

The present study aimed to evaluate, in a large cohort of participants, the ability of the probiotic *L. rhamnosus* CA15 strain, orally administered, to treat BV and co-occurring vaginitis. Previously, Rapisarda and co-workers [16], by conducting a randomized, double-blind, placebo-controlled study on a cohort of 60 women with vaginal dysbiosis, highlighted the ability of the *L. rhamnosus* CA15 strain, orally administered, to significantly improve both clinical signs and symptoms and to modulate the vaginal microbiota. In this context, Rapisarda and colleagues [16] revealed a significant reduction in co-occurring pathogens (e.g., *Gardnerella*, *E. coli*, staphylococci, and *Candida* spp.) and an increase in the cell density of lactobacilli both 10 days after the start of the treatment and 30 days post- treatment.

In line with this evidence, the present study confirmed the ability of the *L. rhamnosus* CA15 strain to balance the vaginal microbiota even 30 days post-treatment, demonstrating a lasting effect over time. In fact, a further increase in lactobacilli and a concomitant decrease in enterococci, staphylococci, and *Gardnerella* spp. were observed 30 days post-treatment. This feature could be associated with the strong adhesion properties and the ability to co-aggregate with pathogens, exhibited in vitro [15], thereby preventing their adhesion to vaginal epithelial cells and reducing colonization. The CA15 strain is also able to produce high amount of lactic acid, which, by lowering the vaginal pH, creates an unfavorable environment for pathogenic microorganisms. Furthermore, the ability to synthesize exopolysaccharides (EPSs) may contribute to the exerted antimicrobial activity while the biofilm formation could enhance its persistence in the vaginal ecosystem. Along with microbiological parameters, the *L. rhamnosus* CA15 strain, administered for 10 days, positively affected both clinical signs and symptoms caused by several pathogens, which are involved in BV and vaginal co-infections, as previously reported by Xiao and co-workers [30]. In the last years, great attention was focused on the increasing prevalence of mixed vaginal infections, a syndrome combining symptoms of different pathogenic processes, mediated by at least two types of vaginal pathogens that cause vaginal inflammation. Similar results were achieved by Vaccalluzzo and co-workers [31], who evaluated the efficacy of the *L. rhamnosus* TOM 22.8 strain, orally administered for 10 days, to restore the physiological conditions of the vaginal microbiota in women with BV and mixed vaginal dysbiosis. The authors, in fact, revealed a significant improvement of all the vaginal signs and symptoms both 10 days and 30 days after the start of the treatment [31]. A single-arm, uncontrolled open-label trial evaluating the blend SYNBIO^®^, a combination of *L. rhamnosus* IMC501^®^ and *Lacticaseibacillus paracasei* IMC502^®^, revealed that the oral administration of the probiotic blend for 15 days determined a statistically significant decrease in itching and vulvovaginal erythema/edema. The severity score attributed to leucorrhea showed an increase, whereas no significant effects on burning and subjective vaginal discomfort were observed. Moreover, seven days after the end of the treatment, both itching and vulvovaginal erythema/edema were characterized by an increasing trend, reaching values similar to those observed at baseline [32].

The evidence of the present study clearly demonstrates the ability of the *L. rhamnosus* CA15 strain to support the reestablishment of a balanced vaginal microbiota. Actually, several clinical trials evaluated the effect of oral probiotics after or during antifungal or antimicrobial treatments, sometimes with conflicting results. In this context, *L. rhamnosus* GR-1 and *Limosilactobacillus reuteri* RC-14 have been extensively assessed as female orally consumed probiotics, although evidence for their oral administration efficacy in the prevention and treatment of vaginal infection conditions, such as BV, remains highly debated [33]. Recently, Zhang and co-workers [34], evaluating the effectiveness of metronidazole and oral *L. rhamnosus* GR-1 and *L. reuteri* RC-14 probiotics, adjunct to metronidazole in the treatment of bacterial vaginosis, revealed that the oral probiotic adjunctive treatment did not increase the cure rate of Chinese BV patients compared to metronidazole alone [34]. Consistent with this study, the rates of BV did not differ between the probiotic and placebo groups after oral administration of *L. rhamnosus* GR-1 and *L. reuteri* RC-14 to pregnant women. Additionally, there were no differences in the α-diversity or the composition of the vaginal microbiota between or within the probiotic and placebo groups at different time points [35], nor in the cytokine and chemokine levels, as shown by Yang and colleagues [36]. Differently, the efficacy of these two probiotic strains was demonstrated in Black Africans and Brazilians, among others [37,38]. A randomized, double-blind, placebo-controlled trial conducted in a cohort of 125 black African premenopausal women diagnosed with BV revealed that the administration of metronidazole (500 mg) twice daily for 7 days, plus oral *L. rhamnosus* GR-1 and *L. reuteri* RC-14 twice daily for 30 days, determined negative sialidase and a shift in Nugent scores to normal values. Few patients, subjected to probiotic administration, experienced mild irritative symptoms, a weakly positive sialidase score, and an intermediate Nugent score. In addition, at the end of this study, a higher count of *Lactobacillus* spp. was observed in vaginal samples from probiotic-treated subjects compared to controls [37]. The hypothesis that the probiotics *L. rhamnosus* GR-1 and *L. reuteri* RC-14 might provide an adjunct to antimicrobial treatment and improve cure rates of BV was evaluated by Martinez and co-workers [38] on sixty-four Brazilian women. Based on the study design, enrolled patients were randomly assigned to receive a single dose of tinidazole (2 g) supplemented with either two placebo capsules or two capsules containing *L. rhamnosus* GR-1 and *L. reuteri* RC-14 for 4 weeks. At the end of treatment, according to the Gram-stain Nugent score, a significantly higher cure rate of BV was revealed in the Placebo group [38].

Among probiotics suitable for oral administration, the *Lactobacillus acidophilus* La-14 (La-14) and *L. rhamnosus* HN001 (HN001) strains showed promising beneficial effects for vaginal health in randomized placebo-controlled clinical trials [39,40,41,42]. Generally, the blend mentioned above was tested in combination with lactoferrin, a glycoprotein with a well-known ability to beneficially affect the vaginal microbiota. Only one clinical trial used the blend of La-14 and HN001 devoid of lactoferrin in premenopausal Caucasian women without vaginal complaints (Nugent score 0–3 and vaginal pH ≤ 4.5) [43]. Although the therapeutic effect of the probiotic blend was not under investigation, this study revealed the ability of the probiotic blend to colonize the vagina, supporting a stable and healthy commensal vaginal microbiota, and to significantly decrease specific immune markers contributing to immunological homeostasis [43].

It is well known that symptomatic vaginal dysbiosis may have a negative impact on quality of life, causing embarrassment, stigmatization, anxiety, and low work productivity [44,45,46,47,48]. Moreover, it is also recognized that recurrent urogenital infections also contribute to stress and can influence mental health [48]. In the present study, by using the WHO-QOL-BRIEF questionnaire, a significant improvement in the quality of life was experienced by women subjected to the *L. rhamnosus* CA15 strain’s administration. In particular, improvements in the environment, social relations, and physical health domains as well as in the overall perception of quality of life were revealed, confirming the results previously reported by Rapisarda and co-workers [16]. Interestingly, in the present study, a significant improvement in the psychological health domain was also achieved. Actually, few studies evaluated, in women with vaginal dysbiosis, the effects of probiotic administration on the quality of life [31,32,49]. Vaccalluzzo and co-workers [31], through the Short Form-36 (SF-36) questionnaire, demonstrated the ability of the *L. rhamnosus* TOM 22.8 strain to improve physical and emotional limitations as well as social functioning. Similarly, Ang and colleagues [49], by using the vulvovaginal symptom questionnaire (VSQ), revealed that the administration of a blend of lactobacilli (SynForU-HerCare) in pregnant women determined a reduction in emotional and social stress. Differently, Pino and co-workers [32], by using the WHO-QOL-BRIEF questionnaire, revealed that the oral administration of SYNBIO^®^ did not provide any significant improvement in the quality of life.

This study was limited mainly by the short follow-up period and the lack of participant stratification based on the diagnosed vaginal infection. Another limitation of the present study is that the microbiological analysis was focused only on *Gardnerella* spp. as the main marker of BV. In fact, several other fastidious anaerobes (e.g., *Atopobium*, *Prevotella*, and *Mobiluncus*), which also contribute to the BV dysbiosis, were not detected in the present study, due to their fastidious nutritional requirements, which may not be adequately supported by culture media and anaerobic conditions used for plate counting. Apart from this, ongoing studies are focusing on the detection of the *L. rhamnosus* CA15 strain in both vaginal and fecal samples, which could provide valuable insights into its therapeutic potential.

## 5. Conclusions

In conclusion, the administration of the *Lacticaseibacillus rhamnosus* CA15 (DSM 33960) probiotic strain can be considered an effective and safe strategy to re-establish the balance of the vaginal microbiota, to manage clinical signs and symptoms, and to improve the quality of life of women with BV and co-occurring vaginal infections.

## Figures and Tables

**Table 1:** Baseline demographic characteristics of recruited subjects allocated to Active and Placebo groups.

Demographic Characteristics	Active Group (n = 100)	Placebo Group (n = 100)	*p*-Value
Age	32.58 ± 5.74	32.13 ± 6.52	0.7727
Body mass index			
<18.5	4	5	0.7369
18.5–24.9	81	81	
25–29.9	8	8	
≥30	7	6	
Smoking habits	24	22	0.8667
Contraceptive use			
Oral	12	15	0.6796
Barrier	28	30	0.8762
Others	36	34	0.8822
History of vaginal dysbiosis	61	58	0.7733
Sexual activity	68	71	0.7588

**Table 2:** Baseline clinical characteristics of recruited subjects allocated to Active and Placebo groups.

Clinical Characteristics		Active Group (n = 100)	Placebo Group (n = 100)	*p*-Value
Signs and symptoms	Leucorrhea	95	97	0.7209
	Burning	92	89	0.6306
	Itching	79	82	0.7214
	Vulvovaginal erythema/edema	90	88	0.8217
	Subjective vaginal discomfort	95	97	0.7209
Amsel criteria	Homogenous vaginal discharge	78	80	0.8623
	Clue cell presence	67	70	0.7609
	Positive amine test	68	70	0.8785
	Vaginal pH > 4.5	86	87	1.0000
Nugent score	0–3	0	0	0.4738
	4–6	3	5	
	7–10	97	95	
Lactobacillary grade	I	0	0	0.4719
	II	8	11	
	III	92	89	

**Table 3:** Vaginal microbiota composition at baseline (T0), 10 days (T1), and 30 days (T2) after the end of the treatment evaluated by plate count.

Microbial Groups	Active Group (n = 100)						Placebo Group (n = 100)					
	T0	T1	T2	*p*-Value T0 vs. T1	*p*-Value T0 vs. T2	*p*-Value T1 vs. T2	T0	T1	T2	*p*-Value T0 vs. T1	*p*-Value T0 vs. T2	*p*-Value T1 vs. T2
*Lactobacillus* spp.	3.49 ± 0.08	7.19 ± 0.71	7.21 ± 0.76	3.89 × 10^−18^ *	3.89 × 10^−18^ *	0.0033 *	3.51 ± 0.12	3.52 ± 0.14	3.53 ± 0.34	0.1760	0.0039 *	0.0216 *
*Enterococcus* spp.	4.93 ± 0.57	2.17 ± 0.99	2.14 ± 0.86	3.89 × 10^−18^ *	3.89 × 10^−18^ *	0.0026 *	4.90 ± 0.59	4.99 ± 0.57	4.98 ± 0.58	0.0001 *	0.0000 *	0.7258
*Staphylococcus* spp.	3.70 ± 0.36	1.83 ± 0.22	1.76 ± 0.24	3.89 × 10^−18^ *	3.89 × 10^−18^ *	0.0023 *	3.69 ± 0.29	3.78 ± 0.32	3.79 ± 0.33	0.0000 *	0.0000 *	0.0237 *
*Gardnerella* spp.	4.41 ± 0.50	1.93 ± 0.47	1.92 ± 0.21	3.89 × 10^−18^ *	3.89 × 10^−18^ *	0.0005 *	4.43 ± 0.44	4.53 ± 0.36	4.44 ± 0.43	0.0007 *	0.9014	0.0098 *
*Candida* spp.	3.83 ± 0.29	1.32 ± 0.81	1.34 ± 0.78	3.89 × 10^−18^ *	3.89 × 10^−18^ *	0.6996	3.87 ± 0.42	3.95 ± 0.41	3.92 ± 0.43	0.0006 *	0.0510	0.8231
*Escherichia coli*	4.02 ± 0.68	1.11 ± 0.82	1.09 ± 0.81	3.89 × 10^−18^ *	3.89 × 10^−18^ *	0.1492	4.17 ± 0.48	4.05 ± 0.52	4.11 ± 0.54	0.0000 *	0.0890	0.0078 *

Data are shown as mean log_10_ cfu/mL and standard deviation. * Intra-group statistical significance, at *p* < 0.05, based on Wilcoxon Signed-Rank Test.

**Table 4:** Inter-group differences, at T1 and T2 sampling times, evaluated by Analysis of Covariance (ANCOVA) after baseline (T0) normalization, related to the composition of the vaginal microbiota composition.

Microbial Group	Sampling Time	ANCOVA	
		Effect ^†^	*p*-Value
*Lactobacillus* spp.	T1	3.6978	6.23 × 10^−117^ *
	T2	3.6905	8.40 × 10^−105^ *
*Enterococcus* spp.	T1	−2.8436	1.87 × 10^−72^ *
	T2	−2.8546	5.31 × 10^−78^ *
*Staphylococcus* spp.	T1	−1.9538	6.66 × 10^−118^ *
	T2	−2.0379	4.44 × 10^−118^ *
*Gardnerella* spp.	T1	−2.5927	1.09 × 10^−116^ *
	T2	−2.5226	5.11 × 10^−123^ *
*Candida* spp.	T1	−2.6082	1.58 × 10^−75^ *
	T2	−2.5624	2.95 × 10^−74^ *
*Escherichia coli*	T1	−2.8240	8.47 × 10^−95^ *
	T2	−2.9056	9.29 × 10^−93^ *

^†^ Effect of the treatment on microbiota composition changes, after baseline normalization; * statistical significance at *p* < 0.05.

## Data Availability

The original contributions presented in this study are included in the article/Supplementary Material. Further inquiries can be directed to the corresponding author.

## Supplementary Materials

The following supporting information can be downloaded at https://www.mdpi.com/article/10.3390/microorganisms13122651/s1, Table S1. Adverse events, capsule adherence, and pregnancy during this study. Table S2. Clinical signs and symptoms reported in both Active and Placebo groups at baseline (T0), 10 days (T1), and 30 days (T2) after the end of the treatment. Data are reported as mean values related to the score obtained by the assessment of the intensity of signs and symptoms and standard deviation. Table S3. Inter-groups differences, related to the mean score values obtained by the assessment of the intensity of clinical signs and symptoms. Table S4. Amsel criteria evaluated in both Active and Placebo groups at baseline (T0), 10 days (T1), and 30 days (T2) after the end of the treatment. Data are reported as average frequency. Table S5. Inter-group differences, related to the number of patients satisfying the Amsel criteria, based on logistic regression analysis. Table S6. Nugent score evaluated in both Active and Placebo groups at baseline (T0), 10 days (T1), and 30 days (T2) after the end of the treatment. Data are reported as number of subjects allocated to each score (0–3, normal; 4–6, intermediate; 7–10, dysbiotic). Intra-group differences were evaluated by Wilcoxon Signed-Rank Test for paired data; inter-group differences, at T1 and T2 sampling times, were evaluated by Analysis of Covariance (ANCOVA), after baseline (T0) normalization. Table S7. Lactobacillary grade (LBG) evaluated in both Active and Placebo groups at baseline (T0), 10 days (T1), and 30 days (T2) after the end of the treatment. Data are reported as number of subjects allocated to each grade (LBG I, normal; LBG II, mixed flora; LBG III, suppression of lactobacilli). Intra-group differences were evaluated by Wilcoxon Signed-Rank Test for paired data; inter-group differences, at T1 and T2 sampling times, were evaluated by Analysis of Covariance (ANCOVA), after baseline (T0) normalization. Table S8. Quality of life (QoL) evaluated through the WHOQOL-BREF questionnaire, assessed at baseline (T0) and 30 days (T2) after the end of the treatment.

## Abbreviations

The following abbreviations are used in this manuscript:

ANCOVA	Analysis of Covariance
AV	Aerobic vaginitis
BV	Bacterial vaginosis
cfu	Colony-forming units
COSORT	CONsolidated Standards Of Reporting Trials
EPS	Exopolysaccharide
HIV	Human immunodeficiency virus
HPV	Human papillomavirus
HSV-2	Herpes simplex virus 2
LBG	Lactobacillary grade
MV	Mixed vaginitis
PID	Pelvic inflammatory disease
QoL	Quality of life
SPIRIT	Standard Protocol Items: Recommendation for Interventional Trials
STIs	Sexually transmitted infections
TLRs	Toll-like receptors
VSQ	Vulvovaginal symptom questionnaire
VVC	Vulvovaginal candidiasis
WHOQL-BREF	World Health Organization Quality of Life–BREF
WMA	World Medical Association
