# Supplementary material for: Oral Administration of the Probiotic Lacticaseibacillus rhamnosus CA15 in a Large Cohort of Women with Bacterial Vaginosis and Mixed Vaginitis: Clinical Evidence from a Randomized, Double-Blind, Placebo-Controlled Study
Source: Microorganisms. 2025 Nov 21;13(12):2651. doi: 10.3390/microorganisms13122651 (PMC12734845; doi:10.3390/microorganisms13122651)
Supplement: Supplementary file 1 [file microorganisms-13-02651-s001.zip › microorganisms-3943402-supplementary.pdf]

Supplementary Material

Oral Administration of the Probiotic *Lacticaseibacillus rhamnosus* CA15 in a Large Cohort of Women with Bacterial Vaginosis and Mixed Vaginitis: Clinical Evidence from a Randomized, Double-Blind, Placebo-Controlled Study

Alessandra Pino [1,2], Amanda Vaccalluzzo [1], Stefano Cianci [3], Marco Palumbo [4], Giuseppe Caruso [4], Cinzia Caggia [1,2] and Cinzia L. Randazzo [1,2,*]

[1] Department of Agriculture, Food and Environment, University of Catania, 95123 Catania, Italy; alessandra.pino@unict.it (A.P.); amanda.vaccalluzzo@unict.it (A.V.); ccaggia@unict.it (C.C.)
[2] ProBioEtna SRL Spin off of the University of Catania, 95123 Catania, Italy
[3] Unit of Gynecology and Obstetrics, Policlinico "G. Martino", Department of Human Pathology of Adult and Childhood "G. Barresi", University of Messina, 98125 Messina, Italy; stefano.cianci@unime.it.
[4] Department of General Surgery and Medical Surgical Specialties, Gynaecological Clinic, School of Medicine, University of Catania, 95123 Catania, Italy; mpalumbo@unict.it (M.P.); giu.caruso97@gmail.com (G.C.)
* Correspondence: cranda@unict.it

Table S1. Adverse events, capsule adherence, and pregnancy during the study.

Parameter	Active Group (n=100)	Placebo Group (n=100)	*p*-Value
**Adverse events (AEs)**			
Mild abdominal paint	3	5	n.s.
Nausea	12	7	n.s.
Flatulence	8	9	n.s.
Severe AEs	0	0	n.s.
Capsule adherence (%)	100	100	n.s.
Pregnancy the during study	0	0	n.s.

n.s.: not statistically significant.

Table S2. Clinical signs and symptoms reported in both Active and Placebo groups at baseline (T0), 10 days (T1) and 30 days (T2) after the end of the treatment. Data are reported as mean values related to the score obtained by the assessment of the intensity of signs and symptoms and standard deviation.

Clinical Signs and Symptoms	Active Group (n=100)						Placebo Group (n=100)					
	T0	T1	T2	*p*-Value T0 vs T1	*p*-Value T0 vs T2	*p*-Value T1 vs T2	T0	T1	T2	*p*-Value T0 vs T1	*p*-Value T0 vs T2	*p*-Value T1 vs T2
Leucorrhoea	1.9 ± 0.88	0.58 ± 0.75	0.49 ± 0.63	8.09×10^{-16}*	4.28×10^{-16}*	0.4638	1.87 ± 0.84	1.9 ± 0.80	1.87 ± 0.80	0.9295	0.9537	0.8290
Burning	1.58 ± 0.81	0.47 ± 0.63	0.42 ± 0.57	1.48×10^{-15}*	1.44×10^{-14}*	0.6129	1.55 ± 0.86	1.64 ± 0.86	1.63 ± 0.82	0.6781	0.7730	0.5052
Itching	1.49 ± 0.96	0.43 ± 0.54	0.43 ± 0.56	2.26×10^{-14}*	2.25×10^{-14}*	0.8663	1.53 ± 0.94	1.53 ± 0.95	1.52 ± 0.92	1.0000	0.8801	0.8621
Vulvo-vaginal erythema/oedema	1.51 ± 0.88	0.55 ± 0.66	0.46 ± 0.54	8.29×10^{-13}*	1.28×10^{-13}*	0.2370	1.45 ± 0.89	1.39 ± 0.86	1.34 ± 0.84	0.5832	0.7244	0.9956
Subjective vaginal discomfort	2.2 ± 0.91	0.39 ± 0.63	0.31 ± 0.49	4.95×10^{-18}*	9.43×10^{-18}*	0.3663	2.2 ± 0.86	2.25 ± 0.82	2.19 ± 0.83	0.6992	0.5046	0.2681

*Intra-group statistical significance at $p<0.05$.

Table S3. Inter-groups differences, related to the mean score values obtained by the assessment of the intensity of clinical signs and symptoms. .

Clinical Signs and Symptoms	Sampling Time	Active Group (n=100)	Placebo Group (n=100)	ANCOVA	
				Effect[†]	*p*-Value
Leucorrhoea	T1	0.58 ± 0.75	1.9 ± 0.80	-1.33	1.57×10^{-27}*
	T2	0.49 ± 0.63	1.87 ± 0.80	-1.38	1.16×10^{-30}*
Burning	T1	0.47 ± 0.63	1.64 ± 0.86	-1.17	4.07×10^{-23}*
	T2	0.42 ± 0.57	1.63 ± 0.82	-1.21	2.25×10^{-25}*
Itching	T1	0.43 ± 0.54	1.53 ± 0.95	-1.08	1.07×10^{-27}*
	T2	0.43 ± 0.56	1.52 ± 0.92	-1.07	1.79×10^{-26}*
Vulvo-vaginal erythema/edema	T1	0.55 ± 0.66	1.39 ± 0.86	-0.87	3.01×10^{-18}*
	T2	0.46 ± 0.54	1.34 ± 0.84	-0.89	1.51×10^{-17}*
Subjective vaginal discomfort	T1	0.39 ± 0.63	2.25 ± 0.82	-1.86	8.21×10^{-54}*
	T2	0.31 ± 0.49	2.19 ± 0.83	-1.88	8.22×10^{-56}*

[†]Effect of the treatment on clinical signs and symptoms changes, after baseline normalization. *Statistical significance at $p<0.05$.

Table S4. Amsel criteria evaluated in both Active and Placebo groups at baseline (T0), 10 days (T1) and 30 days (T2) after the end of the treatment. Data are reported as average frequency.

**Amsel Criteria**	**Active Group (n=100)**						**Placebo Group (n=100)**					
	**T0**	**T1**	**T2**	***p*-Value T0 vs T1**	***p*-Value T0 vs T2**	***p*-Value T1 vs T2**	**T0**	**T1**	**T2**	***p*-Value T0 vs T1**	***p*-Value T0 vs T2**	***p*-Value T1 vs T2**
Homogenous vaginal discharge	78	28	16	1.08×10^{-11}*	9.40×10^{-15}*	0.0059*	80	78	77	0.7893	0.7353	1.00
Clue cell presence	67	11	13	1.24×10^{-12}*	7.79×10^{-12}*	0.6830	70	68	69	0.8026	1.00	1.00
Positive amine test	68	13	9	8.52×10^{-13}*	1.11×10^{-13}*	0.3864	70	72	70	0.7237	0.7518	0.6170
Vaginal pH > 4.5	86	9	2	4.67×10^{-18}*	1.35×10^{-19}*	0.0455*	87	87	89	0.8231	0.8312	0.7728

*Intra-group statistical significance at $p<0.05$.

Table S5. Inter-groups differences, related to the number of patients satisfying the Amsel criteria, based on logistic regression analysis. .

Clinical Signs and Symptoms	Sampling Time	Effect†	*p*-Value
Homogenous vaginal discharge	T1	-2.74	2.9471×10^{-11}*
	T2	-2.87	3.5042×10^{-15}*
Clue cell presence	T1	-3.37	6.6542×10^{-14}*
	T2	-2.74	2.9908×10^{-13}*
Positive amine test	T1	-4.23	4.7347×10^{-13}*
	T2	-4.23	8.5187×10^{-15}*
Vaginal pH > 4.5	T1	-4.27	1.3824×10^{-19}*
	T2	-5.91	1.1877×10^{-14}*

†Treatment effect on the changes in the Amsel criteria over time. *Inter-group statistical significance, at $p<0.05$.

Table S6. Nugent score evaluated in both Active and Placebo groups at baseline (T0), 10 days (T1) and 30 days (T2) after the end of the treatment. Data are reported as number of subjects allocated to each score (0-3, normal; 4-6, intermediate; 7-10, dysbiotic). Intra-group differences were evaluated by Wilcoxon Signed-Rank Test for paired data; inter-group differences, at T1 and T2 sampling times, were evaluated by Analysis of Covariance (ANCOVA), after baseline (T0) normalization.

Nugent Score	Active group (n=100)						Placebo group (n=100)						ANCOVA			
	T0	T1	T2	*p*-value T0 vs T1	*p*-value T0 vs T2	*p*-value T1 vs T2	T0	T1	T2	*p*-value T0 vs T1	*p*-value T0 vs T2	*p*-value T1 vs T2	T1		T2	
													Effect†	*p*-value	Effect†	*p*-value
0-3 (Normal)	0	98	100				0	0	0							
4-6 (Intermediate)	3	2	0	1.44×10^{-22}*	6.18×10^{-23}*	0.6962	5	3	2	0.7120	0.5763	0.8437	-1.9498	1.05×10^{-159}*	-1.9797	2.62×10^{-199}*
7-10 (Dysbiotic)	97	0	0				95	97	98							

†Effect of the treatment on Nugent score changes, after baseline normalization. *Inter-group and intra-group statistical significance, at $p<0.05$.

Table S7. Lactobacillary grade (LBG) evaluated in both Active and Placebo groups at baseline (T0), 10 days (T1) and 30 days (T2) after the end of the treatment. Data are reported as number of subjects allocated to each grade (LBG I, normal; LBG II, mixed flora; LBG III, suppression of lactobacilli). Intra-group differences were evaluated by Wilcoxon Signed-Rank Test for paired data; inter-group differences, at T1 and T2 sampling times, were evaluated by Analysis of Covariance (ANCOVA), after baseline (T0) normalization.

LBG	Active group (n=100)						Placebo group (n=100)						ANCOVA			
	T0	T1	T2	*p*-value T0 vs T1	*p*-value T0 vs T2	*p-value* T1 vs T2	T0	T1	T2	*p*-value T0 vs T1	*p*-value T0 vs T2	*p*-value T1 vs T2	T1		T2	
													Effect†	*p*-value	Effect†	*p*-value
LBG I	0	85	93				0	0	0							
LBG II	8	7	5	5.46×10^{-20}*	4.47×10^{-21}*	0.0698	11	6	3	0.3932	0.1469	0.5832	-1.7121	2.51×10^{-68}*	-1.8842	1.21×10^{-111}*
LBG III	92	8	2				89	94	97							

†Effect of the treatment on LBG changes, after baseline normalization; *Intra-group and inter-group statistical significance, at $p<0.05$.

Table S8. Quality of life (QoL) evaluated through the WHOQOL-BREF questionnaire, assessed at baseline (T0) and 30 days (T2) after the end of the treatment.

	Time	Active group		Placebo group		ANCOVA	
		Mean score	*p*-value T0 vs T2	Mean score	*p*-value T0 vs T2	Effect[†]	*p*-value
**Physical Health**	T0	65.39	4.40×10^{-17}*	65.54	0.2086	5.5929	1.00×10^{-17}*
	T2	71.43		65.96			
**Psychological Health**	T0	68.67	0.000819*	68.04	0.7490	1.1207	0.0065*
	T2	69.79		68.08			
**Social Relations**	T0	69.25	1.37×10^{-11}*	68.58	0.4141	8.3055	4.90×10^{-11}*
	T2	76.50		67.75			
**Environment**	T0	65.38	8.14×10^{-17}*	65.81	0.1758	4.3940	3.76×10^{-14}*
	T2	70.34		66.31			
**Overall quality of life**	T0	2.84	4.73×10^{-17}*	2.88	0.8548	1.0268	1.51×10^{-32}*
	T2	3.89		2.89			
**General health**	T0	3.26	0.5832	3.21	0.1398	0.1270	0.0030*
	T2	3.29		3.12			

[†]Effect of the treatment on changes related to each item, after baseline normalization; *Intra-group and inter-group statistical significance at $p<0.05$.
